# Suicidal Ideation in Bereavement: A Systematic Review

**DOI:** 10.3390/bs9050053

**Published:** 2019-05-14

**Authors:** Nicolette Molina, Martin Viola, Madeline Rogers, Daniel Ouyang, James Gang, Heather Derry, Holly G. Prigerson

**Affiliations:** 1 The American Institute for Cognitive Therapy, New York, NY 10022, USA; 2 Cornell Center for Research on End-of-Life Care, Weill Cornell Medicine, New York, NY 10065, USA; mav4001@med.cornell.edu (M.V.); mar2851@med.cornell.edu (M.R.); douyang2012@gmail.com (D.O.); hmd2002@med.cornell.edu (H.D.); hgp2001@med.cornell.edu (H.G.P.); 3 Weill Department of Medicine, Weill Cornell Medicine, New York, NY 10065, USA; jmg2016@med.cornell.edu

**Keywords:** bereavement, caregivers, suicidal ideation, grief, cancer, dementia, accidental overdose, HIV, AIDS, cardiovascular disease

## Abstract

**Background:** Bereavement is associated with impaired mental health, increases in adverse health behaviors, and heightened risk of suicidal ideation, attempts, and death by suicide. The purpose of this literature review was to explore associations between cause of death and suicidal thoughts among bereaved individuals. Our aim was to compare incidence of suicidal ideation by cause of death and identify gaps in this literature to guide future research and clinical intervention. **Methods:** PRISMA-P guidelines were used to structure an electronic literature search in the PsycINFO, MEDLINE, and Web of Science databases. The search focused on English language studies that were published before February 2019 and sought to compare rates of suicidal ideation among bereaved people who lost a loved one to suicide, accidental overdose, cancer, dementia, cardiovascular disease, and HIV/AIDs. **Results:** Nine articles were identified with suicide as cause of death, zero articles for accidental overdose, zero articles for cardiovascular disease, seven articles for cancer, one article for dementia, and one article for HIV/AIDs. Given the limited number of articles generated by our search, a formal meta-analysis was not appropriate. However, a comparison of results did suggest that suicide bereavement was associated with the highest rates of suicide ideation (14.1% to 49%). Stigma, isolation, avoidance behaviors, and psychological distress were associated with suicidal thoughts among bereaved individuals, regardless of the deceased’s cause of death. **Conclusions:** Findings of this literature search revealed significant gaps in the literature, especially regarding thoughts of suicide in bereaved survivors of accidental overdose and cardiovascular disease. Results suggest that multiple causes of death are associated with suicidal ideation in bereavement, but that suicide bereavement may be the cause of death associated with the highest risk of suicidal ideation. More research is needed to understand the ways in which cause of death influences prevalence, risk, and protective factors associated with suicidal thoughts among bereaved individuals.

## 1. Introduction

Bereavement has been shown to be associated with adverse health outcomes, including increased alcohol and tobacco consumption [1] and impairments in physical health, including increased risk of cardiovascular events [2] and heightened risk of mortality [3]. Bereavement also poses risk of adverse mental health outcomes (e.g., anxiety, depression, and prolonged grief disorder (PGD)) [4,5,6]. Elevated risk of suicidal ideation, attempts [7], and suicide [8] may be associated with bereavement as well. For many family members, bereavement may be preceded by a period of informal caregiving for the deceased. Several factors related to informal caregiving have been shown to heighten risk of psychological distress and suicidal ideation among this population. For example, place of death of a family member was associated with post-traumatic stress disorder (PTSD) and PGD in a bereaved sample (specifically among patients with cancer who die in a hospital or ICU) [9]. Additionally, PGD and quality of life/quality of death of the patient may affect bereaved informal caregivers’ mental health [10] and even their degree of suicidal ideation [11]. What remains less well-known and studied is the relationship between the deceased’s cause of death and the bereaved family caregiver’s risk of suicidal ideation.

In addition, stigma has been suggested as a factor implicated in the onset or exacerbation of suicidal thoughts among bereaved survivors of a significant other’s death. Bereaved individuals who reported greater perceived stigma surrounding their loss were more likely to report suicidal ideation [12]. Additionally, stigma and accompanying suicidality may be associated with cause of death, specifically among deaths considered “traumatic” [13].

The aim of this literature review is to compare the evidence and identify gaps in knowledge with respect to the incidence of suicidal ideation among the bereaved based on whether the deceased died by suicide, accidental overdose, cancer, dementia, cardiovascular disease (CVD), or human immunodeficiency virus infection and acquired immune deficiency syndrome (HIV/AIDs), causes of death that collectively represent approximately 50% of all mortality in the United States [14]. These specific causes of death were chosen for two reasons. First, family members bereaved by chronic diseases such as cancer, dementia, and CVD, and HIV/AIDS may have served as informal caregivers before death, while other groups did not, allowing the opportunity to illuminate potential differences between suicidal ideation among caregiving and non-caregiving populations in bereavement. Second, comparing natural causes of death not traditionally associated with stigma (cancer, dementia, CVD) with causes of death historically associated with stigma (accidental overdose, HIV/AIDS, suicide) may elucidate potential effects of stigma on suicidal ideation in bereavement as well. The goal of this review is to identify which bereaved populations are at the greatest need for additional research and intervention to reduce the risk of suicidal thoughts in bereavement.

## 2. Materials and Methods

### 2.1. Search Strategy

Our literature review was guided by the Preferred Reporting Items for Systematic Reviews and Meta-Analysis Protocol (PRISMA-P) statement which provides an evidence-based protocol for systematic reviews and meta-analyses [15]. We conducted an electronic literature search in the PsycINFO, MEDLINE, and Web of Science databases. The eligibility criteria limited our search to English language studies that were published prior to February 2019. The included samples were comprised of individuals bereaved by suicide, accidental overdose, cancer, dementia, HIV/AIDs, or CVD, and there were no specific criteria regarding comparison groups; bereaved samples included a mix of both bereaved informal caregivers and bereaved family members. Our study design criteria included both cross-sectional and longitudinal observational studies that examined rates of suicidal ideation among these bereaved individuals and excluded systematic reviews. Articles were included if the authors reported any endorsement of suicidal ideation regardless of severity. Search terms included “caregiver”, “carers”, “family care”, “grief”, “bereavement”, “suicide risk”, “suicidal ideation”, “suicidal behavior” along with specific terms varying based on cause of death (see Table 1). Additional articles found through citations of the aforementioned articles or reviewer recommendation that met eligibility criteria were also included.

### 2.2. Data Extraction

**Figure 1:**
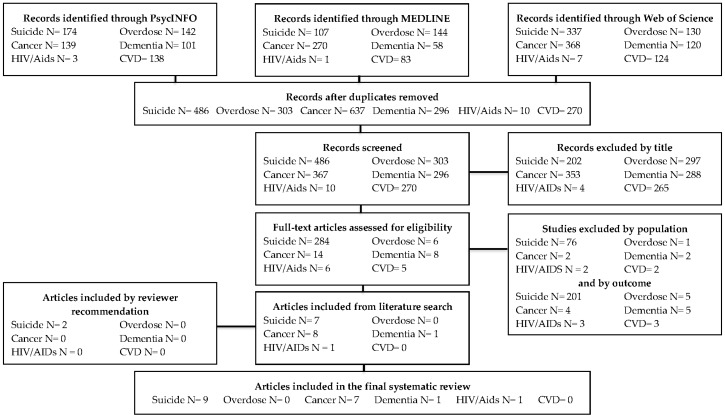
Flow diagram of search process guided by the PRISMA statement [15].

All articles generated by the search were screened by NM, MV, MR, and JG. Articles that did not fit the scope of our review (e.g., studies with samples irrelevant to our review and studies that did not include an outcome of suicidal ideation) were excluded. Data regarding sample, setting, location, outcome variable, main finding, and study limitations from each eligible study was documented. A flowchart with further details on the literature search and search results is shown in Figure 1.

## 3. Results

Results on rates of suicidal ideation in each bereaved population yielded from this search are shown in Table 2. Please note that articles that did not report the rate of suicidal ideation of the bereavement group are not included in Table 2.

### 3.1. Suicide-Bereaved

Our search generated 7 articles on risk of suicidal ideation in suicide bereavement that fit the scope of this review, all published between 2005 and 2018. Two additional studies were included per reviewer recommendation [16,17]. Length of time since suicide varied, with the most recent being 1 month and least recent 10 years since the death by suicide. The sample populations for 4 out of these 9 studies [18,19,20,21] were treatment-seeking study participants.

In a paper from 2005, Mitchell and colleagues found that 10 of 60 recent (17%, within 1 month of suicide) suicide-bereaved individuals endorsed suicidal ideation [18]. Suicide-bereaved individuals with syndromal levels of PGD in this sample were 10 times more likely to report suicidal ideation compared those who did not have syndromal level PGD, even after adjusting for depression (AOR = 9.68, 95% CI = 1.03,−90.41, *p* = 0.046) [18]. A 2010 longitudinal cohort study found 27 out of 122 suicide-bereaved individuals (22%; 13 months post suicide) reported suicidal ideation; ideators were more likely to be a parent or spouse. Ideators were also more likely to have a history themselves of suicide attempt(s) (2/95, 2.1% vs. 5/19, 18.5%, X^2^ (1) = 10.47, *p* = 0.001) and history of depression (19/95, 20.2% vs. 12/19, 46.2%, X^2^ (1) = 7.15, *p* = 0.01) compared to non-ideators [19].

A 2018 study by Williams and colleagues found 43% of suicide-bereaved family members reported suicidal ideation (N= 28; 44.4 months post death). Type of death (specifically, suicide, homicide, or fatal accident), witnessing the death or the death scene, or funeral participation were not associated with endorsement of suicidal ideation [20]. Avoidance symptoms (OR = 2.22, 95% CI, *p* = 0.025) and depression (OR = 1.16, 95% CI, *p* < 0.001), however, were identified as uniquely associated with suicidal ideation. Similar results were found by Kõlves and colleagues in 2019, who compared grief reactions in sudden death bereavement (N = 63) to suicide bereavement (N = 142; 6 months post death) [21]. 14.1% of the suicide-bereaved and 12.7% of the sudden death-bereaved in this study had high risk of suicidal ideation (using a cut-off point of 2 for Beck Scale for Suicide Ideation (BSS)); the difference between the two groups was not statistically significant (X^2^ (1) = 0.07, *p* = 0.790). However, Kõlves and colleagues found that bereavement type significantly predicted (*p* < 0.05) stigmatization, responsibility, and shame, with these grief reactions being highest in the suicide-bereaved group [21].

A South Korean study by Song et al., found 26.7 % (N = 30) of individuals bereaved by the suicide of a family member experienced suicidal ideation in the past year [22]. Additionally, authors found that individuals who lost a family member were 4.5 time more likely to have suicidal ideation in the past year compared to individuals who had not lost a family member to suicide (N = 885) [22]. Ideation was measured by responses of “never”, “sometimes” or “seriously” to the question: “*Have you ever seriously contemplated suicide in the past one year?”* Time since suicide was not reported for this sample. A Portuguese study by Santos et al. found 42% of bereaved family members endorsed suicidal ideation, compared to 5% of the control group, a community sample who did not experience the suicide of a family member [23]. Ideation was measured by the Suicide Ideation Questionnaire with scores at or above the cut-off point of 41 indicating endorsement [24]. More than half the bereavement sample experienced the death of a family member by suicide more than 3 years ago at the time of participation.

A similar percentage of suicidal ideation was found in suicide-bereaved family members in a study by Pitman and colleagues. They found that 49% (n = 614; mean time since suicide 5.1 years) of individuals endorsed suicide ideation, as indicated by responding yes to “*Have you ever thought of taking your life, even though you would not actually do it?”* [16]. Cerel and colleagues found 27.3% of suicide-bereaved family members in their sample (n = 68; 12 months since suicide) endorsed ideation as indicated by responses to “*During the past 12 months did you ever seriously think about committing suicide?*” [17].

Only one study, conducted by de Groot and colleagues in the Netherlands, looked at the course of suicide bereavement longitudinally [25]. The authors found 39/153 (26%) suicide-bereaved relatives endorsed suicidal ideation 2.5 months after suicide; however 8 months after suicide, reports of suicidal ideation fell to 6/68 (9%), but the authors did not analyze if these differences were statistically significant; however, bereaved participants in this study reported less general distress over time [25].

### 3.2. Accidental Drug Overdose-Bereaved

Our search on suicidal ideation among individuals bereaved by accidental overdose yielded no results. However, one excluded article generated by this search provided a general overview of grief difficulties and bereavement outcomes experienced by this population due to stigmatization associated with drug use [26].

### 3.3. Cancer-Bereaved

This literature search produced seven studies investigating suicidal ideation among family members of individuals deceased by cancer, all of whom reported to be informal caregivers for the deceased. Abbott, Prigerson, and Maciejewski found prevalence rates of suicidal ideation in family caregivers of cancer patients to be 12% (n = 15) pre-loss and 16.5% (n = 21) post-loss in a multi-site study [11]. The authors also reported that the poorer family members rated the quality of life at the end-of-life of the deceased, the greater risk these individuals were at for suicidal ideation, even when adjusting for pre-loss suicidal ideation (AOR = 0.79, *p* = 0.023). Additional analyses of the same data set by the same authors found family caregivers’ perception of quality of death was significantly associated with suicidal ideation as well, after adjusting for pre-loss suicidal ideation, relationship to the patient, and education (AOR 1.26, *p* = 0.022) [27]. A South Korean study found that compared to a general population sample, bereaved family caregivers of cancer patients more frequently experienced depressive moods (33.1% vs. 12.5%, *p* < 0.001) and suicidal ideation (31.4% vs. 16.4%, *p* < 0.001) [28]. Controls were from a national health and nutrition study conducted in South Korea and propensity matched to reduce confounding effects. Peteet and colleagues also published brief case studies of 5 individuals who reported suicidal ideation due to the impending death of a family member from cancer and provided recommendations for clinical management in the hospital setting [29]. The authors recommended that when family members express suicidal thoughts or behaviors as the result of anticipatory grief, immediate assessment and coordination with mental health clinicians is necessary.

A very limited amount of research has found that cancer bereavement may put individuals at equal or lower risk of suicidal ideation compared to bereavement by other causes. A study comparing individuals bereaved by cancer, HIV/AIDS and suicide on their responses to the Grief Experience Questionnaire [30] found that cancer-bereaved participants were the least frequent endorsers of a subscale measuring suicidal ideation, self-harm, and anhedonia (mean scores of 1.348 Cancer, 1.940 HIV/AIDS, 2.360 suicide, on 5 item subscale made up of Likert-style questions), but did not determine if these differences were statistically significant nor provided specific prevalence rates of suicidal ideation in the groups [31]. Additionally, a study of children ages 5 to 12 who lost their parents either to cancer or suicide found that those bereaved by suicide were significantly more likely to demonstrate symptoms of depression, but both groups reported suicidal ideation at the same rate as a normative sample, reported by the authors as “approximately a third” without further specification [32].

### 3.4. Dementia-Bereaved

This literature search yielded one study regarding suicidal ideation in bereaved dementia family caregivers. O’Dwyer and colleagues found elevated levels of suicidal ideation in this population, both bereaved and non-bereaved (reported by 16.5% of caregivers with patients currently living at home, 16.3% of caregivers with patients currently living in long-term care, and 13.7% of caregivers who were bereaved within 2 years). However, there were no significant differences in the frequency of suicidal ideation between bereaved and non-bereaved caregivers of dementia patients (X^2^(2) = 0.354, *p* = 0.838). The authors also found symptoms of depression to be a significant predictor of risk of suicidal ideation in a linear regression model [33].

### 3.5. Human Immunodeficiency Virus Infection and Acquired Immune Deficiency Syndrome (HIV/AIDS)-Bereaved

This literature search yielded one 1997 study by Rosengard and Folkman [34] examining a population of gay or bisexual men in a committed relationship with a partner with AIDS. 55% of caregivers in this sample endorsed suicidal ideation at some point during the study, including time points before and after the death of a loved one. Qualifying as a caregiver required living together independently with a partner diagnosed with AIDS who needed assistance with at least two instrumental tasks of daily living. Caregivers in this population reported a higher frequency of suicidal ideation, following the death of their partner than before their death X^2^ (3, n = 253) = 7.99, *p* < 0.05). Suicidal ideation was measured through Likert-style response to the item "has there ever been a time when you thought you would be better off dead?” The authors reported that caregiver burden, lack of social support, feeling socially separated, and reliance on escape-avoidance coping strategies increased the likelihood of suicidal ideation, while optimism was found to be a protective factor (F(54) = 2.23, *p* < 0.0001) [34]. Finally, the authors offered suggestions for clinical intervention, including normalizing the experience of suicidal ideation through open discussion and developing alternative coping strategies.

### 3.6. Cardiovascular Disease-Bereaved (CVD)

This literature search yielded zero articles reporting on the incidence of suicidal ideation in family members bereaved by CVD.

## 4. Discussion

Given the limited number of articles comparing SI of people bereaved from different causes of death, a meta-analysis was not appropriate to summarize risk of SI for different types of loss. Overall, articles included in this review found rates of SI from 16.5% to 31.4% in individuals bereaved by cancer, 13.7% of individuals bereaved by dementia, and 9% to 49% in individuals bereaved by suicide. Rates of SI were not reported for HIV/AIDs, accidental overdose, and CVD-bereaved groups. High rates found in the suicide-bereaved group suggest this is a salient issue to consider in efforts to improve their well-being. It is possible that this is a higher rate than other causes of death, and we believe this may be a result of both inherited and environmental risks for suicide among bereaved survivors to the extent they share the genetic or household risks. Although Song et al. found 31.4% of their bereaved sample of South Korean family caregivers of cancer patients reported SI [28], these results may be related to higher rates of suicide in Korea in general (2.4 times higher than the average suicide rates of other countries) [35] and cultural differences in family caregiving [36] rather than as a function of cancer bereavement, per se.

Suicide-bereaved individuals may grieve differently compared to individuals bereaved by other causes of death [37]. This may be at least partially a function of stigma associated with deaths from suicide. Due to the stigmatization of suicide [38], suicide-bereaved individuals often report feelings of shame, guilt (feeling responsible for the suicide), and rejection [21,39]; an experience that individuals bereaved by other causes of death have been found to report at lower rates [37]. Data collected from a community sample showed suicide-bereaved families were viewed as contributing to the death of their loved on [40] through neglect and failure to provide help to the deceased, suggesting both stigma and blame. In addition to public perceptions of the role families may have in the death of a loved one by suicide, families may also feel a sense of shame and guilt. In a study comparing families bereaved by suicide and accidental death, it was found that families bereaved by suicide experienced guilt, shame, and rejection at higher rates than those bereaved by accidental deaths [41]. Similarly, Pitman and colleagues found that suicide-bereaved individuals experienced more stigma compared to people bereaved by sudden natural death and people bereaved by sudden unnatural death [42]. Furthermore, high perceived stigma in bereavement has shown to be associated with higher likelihood of suicidal thoughts and suicide attempts, compared to bereaved individuals with lower scores of perceived stigma [12]. Results from our search indicate that family members bereaved by HIV/AIDs [31] and accidental overdose also report high rates of stigmatization [26]. Future research should examine how risk factors interact in bereaved populations where cause of death is highly stigmatized, to create and implement appropriate interventions for suicide prevention.

While beyond the scope of this review, families bereaved by suicide have been found to attempt [16,17,43] and die by suicide [43,44,45] at higher rates than families bereaved by other causes of death. Current theories of suicide support feelings of isolation and burdensomeness, combined with the capability and access to lethal means, will increase an individual’s risk of suicide [46]. Thus, the perceived stigma and isolation felt by suicide-bereaved family members, in addition to genetic risks for suicide [47], may help explain these findings.

Eight studies included in our review included informal caregiving populations [11,27,28,29,30,31,32,33]. These articles found rates of SI from 16.5% to 31.4% in informal caregivers bereaved by cancer [11,28] and 13.7% in informal caregivers bereaved by dementia [33]. Informal caregiving is well-established as an independent risk factor for poor mental health [48] and SI [11,33] and the limited results of this literature search show that these risks may continue into bereavement. Dementia-bereaved individuals, however, exhibited higher rates of SI pre-loss compared with post-loss [33]. These results suggest that changes in SI from active caregiving to bereavement may be linked with cause of death, and possibly confounders such as the age, gender, and kinship relationship between family caregiver and deceased as well. More research is needed to understand how factors specific to the caregiving process, such as medical care, financial burden, or cultural beliefs, contribute to SI in family member bereavement.

Results from our review indicated several gaps in the literature. First, far greater standardization is needed for quantifying SI and behavior for more accurate comparison across studies. In 19 studies, 10 different measures of SI were used (see Table 2). Additionally, many of the studies were conducted on primarily white/Caucasian race samples. More research is needed on ethnically and racially diverse populations. Studies retrieved in our review were also overwhelmingly treatment-seeking bereaved family members. There is a need for studies that include bereaved populations who do not seek help. Caregivers were underrepresented in the results generated by our systematic review. Given the risk associated with caregiving, further research is needed to investigate the presence of SI in this population. Lastly, women were also over-represented in studies; although women are at higher risk of SI and historically more likely to be informal caregivers [49], men are overwhelmingly more likely to die by suicide [50]. Future research should attend to adequate inclusion of men in study sampling.

## 5. Conclusions

Cause of death may be related to SI in surviving bereaved caregivers and family members, though few studies compared rates between causes of death directly. Specific risk factors and rates of suicide ideation reported in suicide bereavement points to a need to examine SI in this population. There was also a paucity of research on SI in bereaved relatives of those with accidental drug overdose, despite the increase of overdose-related deaths in the United States seen in recent years [51]. It appears that detection and reduction of stigma, isolation, avoidance behaviors, and psychological distress as manifest in symptoms of PGD or depression or anxiety, would likely reduce rates of SI among bereaved caregivers. The result yielded by this review suggest the need for more research into the influence of cause of death and the role of stigma contribute to SI among bereaved survivors. It appears that both play a role in contributing to elevated suicidal risk among bereaved family members and research is needed to inform the development of interventions to reduce this risk.

## Figures and Tables

**Table 1:** Search strategy by bereavement group.

Cause of Death	Search Terms
Suicide	(suicide bereavement OR bereaved by suicide) AND (family OR carers OR caregivers) AND (suicide risk OR suicidal ideation OR suicide)
Accidental overdose	(accidental overdose bereavement OR bereaved by accidental overdose OR accidental overdose OR substance misuse death bereavement OR bereaved by substance misuse death OR substance misuse death OR overdose bereavement OR bereaved by overdose OR overdose) AND (family OR carers OR caregivers) AND (suicide risk OR suicidal ideation OR suicide)
Cancer	(cancer bereavement OR bereaved by cancer OR cancer) AND (family OR carers OR caregivers) AND (suicide risk OR suicidal ideation OR suicide)
Dementia	(dementia bereavement OR bereaved by dementia OR dementia) AND (family OR carers OR caregivers) AND (suicide risk OR suicidal ideation OR suicide)
HIV/AIDs	(HIV bereavement OR AIDS bereavement OR bereaved by AIDS OR bereaved by HIV) AND (family OR carers OR caregivers) AND (suicide risk OR suicidal ideation OR suicide)
Cardiovascular Disease	(cardiovascular disease bereavement OR bereaved by cardiovascular disease OR cardiovascular disease OR stroke bereavement OR bereaved by stroke OR stroke OR heart disease bereavement OR bereaved by heart disease OR heart disease) AND (family OR carers OR caregivers) AND (suicide risk OR suicidal ideation OR suicide)

**Table 2:** Cause of death and percent of sample reporting suicidal ideation (SI) in bereavement.

Cause of Death	% of Sample Endorsing SI	Time Since Death	Measured by	Population	Citation
**Suicide**	16%	1 month	Beck Depression Inventory (BDI); item 9	N = 60; First-degree relatives and spouses part of a crisis intervention study	Mitchell et al., 2005 [18]
	22%	<8 weeks	BDI; item 9	N = 122; First-degree relatives and spouses	de Groot et al., 2010 [19]
	43%	44.4 months (mean)	BDI; item 9	N= 28; Family members seeking mental health services	Williams et al., 2018 [20]
	14.1% ^a^	6 months	Beck Scale for Suicide Ideation (BSS)	N = 142; Adults, family, and friends of deceased	Kõlves et al., 2019 [21]
	26%	2.5 months	Paykel suicide items (PSI) ^b^	N = 153; First-degree relatives and spouses	de Groot et al., 2013 [25]
	9%	8–10 years	Paykel suicide items (PSI) ^b^	N = 68; First-degree relatives and spouses	de Groot et al., 2013 [25]
	26.7%	Not reported	Endorsement of “Have you ever seriously contemplated suicide in the past one year?” ^c^	N = 30; family members	Song et al., 2005 [22]
	42%	Less than 3 years (n = 20; 21.5%) More than 3 years (n = 73; 78.5%)	Suicidal Ideation Questionnaire (SIQ)	N = 93; family members	Santos et al., 2015 [23]
	49%	5.1 years	Endorsement of “Have you ever thought of taking your life, even though you would not actually do it?” ^d^	N = 614; 48% of sample was blood-related to deceased	Pitman, et al., 2016 [16]
	27.3%	<12 months	Endorsement of “During the past 12 months did you ever seriously think about committing suicide?”	N = 68; family members	Cerel et al., 2005 [17]
**Accidental overdose**	No results found				
**Cancer**	16.5%	6.5 months (median)	Yale Evaluation of Suicidality (YES)	N = 127; Adult informal caregivers from recruited from major metropolitan hospitals	Abbott et al., 2014 [11]
	31.4%	2–6 months	Endorsement of “Have you had suicidal thoughts at any time during the previous year?”	N = 501; Family members of deceased patients from registered from the Korean Terminal Cancer Patient Information System	Song et al., 2012 [28]
**Dementia**	13.7%	<2 years	Suicidal Behaviors Questionnaire—Revised (SBQ—R); item 2 ^e^	N = 566; Adult informal caregivers from the mainly from Australia, Canada, and the US	O’Dwyer et al., 2015 [33]
**HIV/AIDs**	Not reported ^f^				Rosengard et al., 1997 [34]
**Cardiovascular Disease**	No results found				

^a^ % of individuals with high risk of suicidal ideation as indicated by cut-off point of 2 for BSS. ^b^ individuals with a score >8 were considered to have suicidal ideation. ^c^ participant responses were operationalized in two ways: “never/sometimes” or “seriously”. ^d^ participant responses of yes or no to “Have you ever thought of taking your life, even though you would not actually do it?”. ^e^ participant responses of “rarely” or “never” to the item “how often have you thought about killing yourself in the past year?” were classified as negative for suicidal ideation, and those who responded “sometimes” “often” and “very often” were classified as positive for suicidal ideation. ^f^ Rosengard and Folkman reported that 55% of their sample endorsed suicidal ideation at some point during the study, including periods pre- and post-bereavement. For that reason, this percentage is not reported in Table 2.
